# “We wish we had the option”: a qualitative study of women’s perspectives and experiences with contraception in a provincial prison in Ontario, Canada

**DOI:** 10.1186/s40352-024-00269-6

**Published:** 2024-04-12

**Authors:** Reilly Jones, Sasha Lemberg-Pelly, Brigid Dineley, Jessica Jurgutis, Fiona G Kouyoumdjian, Jessica Liauw

**Affiliations:** 1 School of Medicine, Queen’s University Kingston, Ontario, Canada; 2 Department of Family Medicine, McMaster University, Ontario, Canada; 3 Department of Obstetrics and Gynaecology, University of British Columbia, Vancouver, Canada; 4 Departments of Gender and Women’s Studies and Indigenous Learning, Lakehead University, Ontario, Canada

**Keywords:** Contraception, Incarceration, Women’s health

## Abstract

**Background:**

Evidence suggests that women who are incarcerated desire access to contraception while incarcerated, and that this need is not currently being met. Our objective in this study was to explore the perspectives and experiences of women in prisons regarding contraception and contraception access using data from focus groups with women in a provincial prison. We analyzed focus group data collected in a provincial prison in Ontario, Canada using content analysis and a constructivist epistemology.

**Results:**

We conducted three focus groups, each approximately one hour in length. Discussions revolved around (1) knowledge and decision making about contraception, (2) accessing contraception, and (3) ideas for increasing access to contraception in the prison setting. Decision making about contraception was mainly related to concerns about side effects, consistent access to care, impacts on future fertility, and autonomy around decision-making. Participants discussed a wide range of experiences with contraception. Ideas for increasing access to contraception included information sessions, inclusion of discussions about contraception as a component of admission and release planning, and time spent in prison as a crucial juncture for decision-making about contraception.

**Conclusions:**

More qualitative research is needed to better understand the needs of women in prisons related to contraception. The findings of this study suggest that programs should focus on consistency and continuity of access to care, education opportunities, and integration of discussions about contraception into official admission and release procedures.

**Supplementary Information:**

The online version contains supplementary material available at 10.1186/s40352-024-00269-6.

## Background

Women are increasingly incarcerated in both Canada and internationally, reflecting their disproportionate experience of intersecting oppressions related to gender, race, and poverty (Kajstura, 2018). In Canada, this is starkly illustrated by the disproportionately high rate of incarceration of Indigenous women, accounting for 42% of custodial admissions in provincial and territorial correctional facilities and almost half of women in federal prisons, while Indigenous people make up 5% of Canada’s population overall (Malakieh, 2022; Maillet, 2023). In 2018/2019 there were 33,119 female admissions to provincial/territorial custody, and 465 admissions to federal custody overall (Malakieh, 2020). Approximately two thirds of incarcerated women in Canada have one or more children and the majority are of reproductive age, with 58% under 35 years old (Reitano et al., 2015).

Evidence indicates that there is an unmet need for contraception among women in provincial and territorial prisons. A survey from Ontario, Canada found that most incarcerated women were sexually active with a man in the three months prior to incarceration and intended to be sexually active with a man in the months following release (Liauw et al., 2016). The same study found that 80% of women who were sexually active with a male and didn’t want to conceive were not using a reliable form of contraception (Liauw et al., 2016). Research in the US has found that most women in prison would accept contraception if it were offered to them during incarceration (Larochelle et al., 2012), and strikingly, that when women are given access to contraception while incarcerated, they are 15 times more likely to initiate contraception compared to when they are provided with information on community resources to access free contraception following release (Clarke et al., 2006).

While this evidence suggests a strong imperative to enhance contraception access and services in custody, we need further information from the perspectives of women who have experienced incarceration on their experiences with these services, and how they may be improved. Prior studies with women in prison have focused on their perspectives and experiences regarding reproductive healthcare in general (Liauw et al., 2021) and pregnancy and the postpartum period (Cavanagh et al., 2022). One previous study in a US jail explored incarcerated women’s attitudes toward contraception and found that while the vast majority of participants felt contraception should be provided to them, many expressed that they would be hesitant to use it themselves. Reasons cited were negative perceptions of the prison’s health care services, fears concerning birth control safety, a desire for future pregnancy, as well as difficulty with follow-up post-incarceration (Schonberg et al., 2015). It is unclear to what extent the findings of that study may be generalizable to women in other facilities and jurisdictions.

Additional research is warranted to validate and enhance what is known already about women’s interests in contraception and contraceptive care in the prison setting. In this study, we aimed to explore the perspectives and experiences of women in a Canadian provincial prison regarding contraception and contraception access.

## Methods

### Recruitment and data collection

We conducted focus groups in 2018 at a provincial prison in southern Ontario. Provincial prison populations in Canada include women who are awaiting trial or sentencing, or have been sentenced to less than two years in custody (Malakieh, 2020). The majority (60%) of adults in provincial or territorial custody have a length of stay of less than a month, and 30% stay less than a week (Malakieh, 2020). Throughout this article the medical term “female” is used only when in reference to officially reported data. The term women refers to cis- and trans-women, as well as other gender fluid or non-binary people who reside in carceral facilities officially designated for people identified as female at intake. Persons were eligible to participate if they were English-speaking, self-identified women between the ages of 18 and 45, and were able to consent. We recruited participants using posters and announcements. We conducted three focus groups with 5–8 women per focus group, for a total of 21 participants.

Focus groups were facilitated by JJ. This team member is a cis woman, and at the time of the focus groups she was a PhD candidate with a Masters of Arts. She had graduate level training in qualitative methodology and methods, as well as feminist epistemologies. Participants were informed of JJ’s academic affiliation and research background. They were also told about the objectives of the study (i.e., to understand their needs with respect to contraception and reproductive healthcare), as well as how it fit into the context of other work done on this topic by the researchers.

Only the facilitator and participants were present during the information and consent process and for the focus groups. The facilitator obtained written consent for participation and audio recording from each member of all focus groups. During the focus groups, questions and prompts were provided verbally to the participants by the facilitator. No participants refused to participate or dropped out of the study. Each of the focus groups lasted approximately 1 h. Across the three focus groups, discussions included content from the same categories and sub-categories, suggesting saturation (Saunders et al., 2018). Transcripts were not returned to study participants for review (Saunders et al., 2018). The focus group guide is available in Supplementary Table 1.

**Table 1:** Data category and sub-category structure

Category	Sub-Category
Experiences with and decision making about contraception	Experiences with contraception
	Considering side effects and concerns regarding contraception
	Importance of ease of use of contraception
Accessing contraception	Uncertain or inconsistent access to contraception while incarcerated
	Importance of accessible contraception while incarcerated
	Nature of the prison setting as an opportunity to access contraception
Ideas for increasing access to contraception in the prison setting	Contraception as a component of admission and release planning
	Information sessions about reproductive health including contraception

### Data analysis

RJ and SLP performed the data analysis for this study. Both are cis women and have a Master’s in Public Health in Health Promotion, during which they received training in qualitative research methods and methodology. SLP has experience in qualitative research related to universal basic income and access to mental health telemedicine (Singla, 2020; O’Mahen, 2008). At the time of the analysis, RJ was a medical student, and SLP was working in communications related to governmental gender equity initiatives. Neither had any contact with study participants. We employed content analysis and used a constructivist epistemology.

We derived categories from the data (as opposed to a priori) through independent coding by RJ and SLP. The coding matrix was discussed and refined with the research team, and the data were re-coded iteratively until our project team came to consensus regarding the categories. All coding was done using NVivo version 12.

Participants were not given the opportunity to provide feedback on the findings of the study. Participants were not identified during focus group recordings, therefore they are not differentiated between in the presented quotations.

This is the second analysis of data collected during these focus groups. The objective of the previously published paper was to explore women’s experiences and perspectives of reproductive healthcare in prison (Liauw et al., 2021). This research builds on that work by focusing on women’s experiences around contraception specifically. This work has allowed us to characterize this component of these data in a way we were not able to in the previous paper.

## Results

The final coding matrix is shown in Table 1. The number beside each included quote indicates the focus group from which the quote was taken.

### Experiences with and decision making about contraception

#### Experiences with contraception

Different participants reported having used a variety of contraception methods. More than one participant in each focus group described having had experience using barrier methods (condoms), depot medroxyprogesterone acetate (DMPA), the oral contraceptive pill, and the intrauterine device (IUD). Some participants also described using barrier methods, withdrawal, or avoiding penetrative sex:

“Just condoms or like, just no sex, like I choose not to have a lot of sex with people.” [3].

“Just pull out, tell the guy to pull out.” [3].

#### Considering side effects and concerns regarding contraception

Across focus groups, participants repeatedly discussed concerns about potential side effects associated with contraception use such as pain, weight gain, acne, impact on mood, or interference with the experience of sex for their partner.

“I’ve done the pills, but they made me bleed the whole time, and I’ve had an IUD from here, but it caused too much pain, so I had to take it out.” [2].

“I’ve only tried the pill, because you can gain weight on the shot, I heard.” [1].

“And during sexual intercourse, I believe, that your partner feels it [an IUD].” [1].

“… there is birth control that makes you fat, there’s birth control that makes you very depressed, there’s birth control that makes you break out, there’s birth control that doesn’t work very good, or yeah, you forget to take them. It’s just, there’s a whole bunch. And the IUD is, the only one bad thing about it is, you don’t get your period and it’s just a bad habit, I guess, when you do get it taken out and you’ll be starting your period, again, it’s kind of weird.” [3].

Regarding IUDs, participants in one focus group expressed concern about having something in their body:

“I hear good things about it and I believe it might be a good thing, but I just don’t know if I would want something inside of me …” [3].

“I just don’t know if I’d want something inside of me that long.” [3].

A minority of participants were concerned with the use of hormones as contraception, and chose options they perceived as more natural:

“In my younger years, I used the pill, and then after my daughter, I did the needle. And then I just completely stopped taking birth control. I don’t believe in birth control, just because of… all the different symptoms, the hormones messing with the chemicals in my body. I want to be natural and be able to control whether I get pregnant on my own….” [3].

Participants across focus groups were concerned with more long-term health consequences related to contraception, like the perception that certain contraception methods would increase their risk of cancer, or decrease their fertility later in life:

“And then there was a study showing that it [the hormonal IUD] was causing like cancer and stuff like that. Scary, I’m scared to do that.” [3].

“I really think like, they’re really messing with my chances of getting pregnant later on in life by not looking into this contraceptive thing removed. Every day I sit in here, my chances are getting worse, right?” [1].

“I said, I’m kind of scared to take any birth control because I want to have another baby and I had a miscarriage, well, not a miscarriage; I had an abortion eight years ago when my son was two, and I haven’t gotten pregnant since then, and I haven’t been on birth control, so I’m like scared that it would lower my chances of having a baby; so, I’m kind of just worried about taking any birth control that might also affect whether or not I can have a baby in the future. Like, even though I’m, obviously, not ready to have one at this moment because I’m still living this lifestyle, but I don’t want to take something that might destroy my chances of having a baby.” [1].

“Sometimes, like there’s a plan B option, but that’s $50. My sister was using that, like almost like every couple weeks, and I was like it’s not been out enough, stop, we don’t know the repercussions of it later in life…” [1].

#### Importance of ease of use of contraception

Many participants discussed the importance of consistency of use of contraception, and concerns they had about using types of contraception that require daily maintenance:

“I personally chose the IUD, because it has… lower maintenance. The pill, you got to take every day and, you know, you got to make those doctor’s appointments and, honestly, my life outside, it sometimes does not allow me to.” [1].

Challenges associated with adherence made longer-acting contraceptive options more appealing to some participants:

“That’s why I went on Depo-Provera shot, was because I could only have to remember that I see my doctor in three months, and then I can get my Depo-Provera shot again.” [1].

Another participant explained their preference for the progesterone implant over DMPA, since it “lasts two years” as opposed to three months.

Although appealing due to its low maintenance, participants across focus groups worried about having the ability to have IUDs removed and replaced in a timely fashion, especially in a prison setting.

“I tried to get my IUD taken out, because it’s just went by, the over five years, because my son is five and I got it after he was born. So, I realize, like it’s like time to just change it, right, and get a new one, but I can’t do that. I put in a request for the doctor. The doctor is never going to take my old IUD out and replace it because it’s expired, he’s probably going to laugh after that […]. So, I don’t know if I can get sick from an old one.” [1].

For some participants, a lack of access to reproductive healthcare while incarcerated meant that the benefits related to the low maintenance of the IUD were offset by concerns about the long-term impacts of its use in the context of having inconsistent access to reproductive healthcare.

### Accessing contraception

#### Uncertain or inconsistent access to contraception while incarcerated

Although they desired it, some participants said that they believed they would not be able to access contraception while incarcerated, or that there might be limitations to being able to access contraception:

“You can’t ask for anything because you’re not allowed to masturbate, and you’re not having sex.” [3].

“…unless you’ve been taking it before you came to jail, they won’t give it to you.” [3].

“I don’t think it’s ever brought up to us, that’s the bottom line.” [3].

“We might be offered it, like they might put you on birth control here, birth control might be an option, but it’s not talked about, you know, so, it’s not — we’re not familiar with it.” [2].

“…we wish we had the option to get an IUD inserted into us before we left the jail. I don’t think they would have the proper medical equipment here to do that.” [3].

Participants in more than one focus group described uncertainty about their ability to access contraception while incarcerated, for both pregnancy prevention as well as other gynecological health concerns:

“Do they even provide birth control while you’re in here… I was wondering, because some people like to be able to control when they get their period and stuff. I didn’t know if they give it to you while you’re here. I think that birth control in here should be something that is offered, but unless you were asked, they wouldn’t bring it up or offer it to you. I don’t know why. I mean, probably because we’re not in pregnancy, but it helps other than preventing pregnancy.” [2].

“I want birth control, I want to be using birth control, just for the added estrogen, because I don’t have a lot of estrogen, so is that something they can do for me, medically…” [3].

#### Importance of accessible contraception while incarcerated

Participants across focus groups expressed beliefs that access to contraception in custody is important for two reasons. First, they perceived that many women become more fertile during their time in prison because they may be eating more or have stopped using drugs during their stay, and second, after being away from their sexual partner or partners while in prison, many planned on being sexually active with a man soon after their release:

“Especially for a lot of us who have been incarcerated for months at a time, where our body is restoring back to that healthiness… when we get released and it’s easier for us to get pregnant and stuff like that, right. Like our body has gone through detox, if we were using alcohol or narcotics or drugs… We’re eating three meals a day, so our bodies are restoring back to that healthy body we once had, right, so our hormones, everything, like our cycle; like for me, I’ve been using narcotics for like eight, nine, ten years and for the last four or five years, I wasn’t getting my period. I wasn’t getting my menstrual cycles. I just got my first period a couple of weeks ago.” [3].

“When I am on drugs, I don’t get a period and I don’t get pregnant, but when I’m sober, I can get pregnant, like really easily, right.” [2].

“I think that it would be really good because, if they offered birth control, especially like, even though we’re not at high-risk of pregnancy in here, women are super sexually active when they leave here, because they’ve been held for so long. I know so many women who leave a facility or an institution, like here and get pregnant.” [2].

#### Nature of the prison setting as an opportunity to access contraception

While time spent in prison was broadly described as challenging, some participants talked about how for them, and for their peers, their stay in a prison setting represented “a moment to pause,” without the same competing priorities and stresses they had on the outside, including drug use, making it an opportunity to seek out healthcare that they may not have in the community, including contraception. Participants also noted the importance of healthcare providers in the prison, and their potential to improve health even after release.

"People are the healthiest when they’re in here [referring to being in prison]."[1].

“Yeah. Because they’re not using. So, it’s a good start, people to sort of have a moment of pause.” [1].

“I have to take care of myself, as well as I should on the inside because I’m sober and clear minded, right? And I think that the nurses should be taking advantage of that because we’re trying now. If, maybe if they showed care, then we’d want to do it for ourselves on the outside. Stop using drugs because, like you notice, like you can better certain things, right.” [2].

### Ideas for increasing access to contraception

#### Contraception as a component of admission and release planning

When asked for suggestions related to the creation of contraception-related services in prison, participants repeatedly discussed interest in the inclusion of discussions about contraception into release planning or on admission to the facility:

“It’s not brought up to us. We see the nurse and they ask us about our medical thing, it’s not in the question, would you like to, so maybe that would be a consideration to have it on the medical form when we first get admitted, would you like to be put on birth control. And then you can have the option of them asking when we get admitted, or we know that we can, at any time, go to the doctor to find out.” [3].

“It should be part of your release plan, I think, to talk about it.” [2].

#### Information sessions about reproductive health including contraception

Participants across focus groups expressed wanting to have information sessions on reproductive health-related topics including contraception while in prison. Many felt that they didn’t have the information necessary to make an informed decision about contraceptive use or method, and that their time in a prison setting would be a good time to obtain that information:

“Because we only get three to four minutes with the doctor when we see the doctor. The doctor is not going to sit there and go through all the different birth controls and all the different options we have.” [3].

“Imagine how much different everything would be if they offered like, some kind of information session here [on contraception], like offered to work with the doctor to get you on what’s best for you, and then showed you where you can go in the community, […]” [1].

“I really think that the health class is in like, in need.” [1].

“…a class where you come to and I don’t know, you hear about STIs and […], you know what I mean and learn about the reproductive system for females…” [1].

“….also like, [a class to] show you the process of a pregnancy as a female, how your body changes, how you vary mentally, physically, emotionally. Like, you should have all that, you know.” [1].

“Or even on information as to where you can see a doctor on the outside, but if you decide that you might not want to in here, but when you get out, maybe you might want to, you might change your mind.” [2].

“It should be integrated like a group, like a half hour session, just to like, mention about birth control and let other women know that there’s options for them to start it, and everything else can get regulated while they’re in here, so they can have it when they get out.” [2].

[In reference to contraception]: “So, what would be good is if there’s […] a file with all the information… this is medications we have available, which one do you want to be on? And then they can take it back to their cell, they could read up on everything… at least you’re breaking it down… And then by the time you go to go get it done, it’s a lot quicker. You can say, I want this, but I was interested in this, and then the next thing you know, you’re on either one.” [3].

## Discussion

In this study, incarcerated women participating in focus groups described substantial concerns about contraception side effects, long term effects of contraception, and consistent care access. They also identified concrete strategies to improve knowledge and contraception access in the prison setting.

Our findings are largely in line with existing knowledge on experiences and concerns regarding contraception among women experiencing incarceration. One similar qualitative study conducted with incarcerated women in a US jail found that most participants believed that they should have access to contraceptive care, but would hesitate to use such services themselves based on concerns about receiving healthcare in prison in general, challenges related to follow-up in the community, and the perception that contraceptive use could threaten their fertility in the long-term (Schonberg et al., 2015). Another US study found that 50% of incarcerated women surveyed who were interested in contraception were interested in the oral contraceptive pill and 48% desired DMPA (Clarke et al., 2006). 2% talked about wanting IUDs, and participants expressed concern regarding consistent access to care and future fertility. These data are similar to our study findings that women had concerns about contraceptive use in relation to its impact on their future fertility, and these perceptions were especially prevalent in relation to discussions about IUDs and other forms of contraception that necessitate consistent medical follow-up. Also similar to our results, a US study by Oswalt et al. (2010) found that 38.5% of surveyed women wanted to become pregnant after release from jail, and only 57% believed that they would have access to healthcare after release. These data reflect our findings that women’s fertility-related goals were complex and evolving, and that consistency of access to healthcare was perceived as a barrier to achieving both contraception and fertility-related goals.

There are inherent challenges associated with the realization of reproductive rights and autonomy in a setting that is designed to be punitive, and to deny its residents their freedom of movement. Participants’ concerns about the impact of contraception on their ability to become pregnant in the future is situated in the context of historical and contemporary coerced sterilization of incarcerated women, most recently documented in California in 2010 (Johnson, 2013; Winters and McLaughlin, 2019; Roth & Ainsworth, 2015). Beyond overt coercion about contraception, incarcerated women often have their desire for pregnancy pathologized and delegitimized (Ross & Solinger, 2017; Schonberg, 2015). The concern about future fertility among study participants speaks to the importance of addressing all aspects of reproductive justice when conceptualizing contraception-related care and programming for incarcerated people (Schonberg et al., 2015; Ross & Solinger, 2017). This includes the right to have children, the right not to have children, and the right to parent children in a safe and healthy environment (Ross & Solinger, 2017).

Participants’ suggestions for improving access to contraception while incarcerated are also supported by existing research. The sentiment that time spent in prison can be a “moment of pause” to focus on one’s health, has been well documented elsewhere in the literature (Clarke et al., 2006; Liauw et al., 2016; Peart & Knittel, 2020; Cannon et al., 2018; Sufrin et al., 2017). Participants’ suggestions to inform women of the availability of contraception upon admission and prior to release, as well as providing educational sessions on contraception, is also in line with previous research that only 4% of women who expressed interest in initiating contraception did so if they were connected with a free clinic post-release, as opposed to 39% among those who had it offered to them while they were incarcerated (Clarke et al., 2006).

Limitations of this study include firstly that the term ‘women’ was used during recruitment of participants. We understand that females who may want to access contraception are not limited to those who identify as women, and not all those who identify as women have contraception needs. This terminology limits the extent to which our study reflects the diversity of people held in prison settings, and adheres to the false gender binary on which the prison system is currently based. Further, aside from age we collected little demographic information about participants, which limited our ability to characterize our study population. This decision was in part due to the challenging nature of conducting qualitative research in a prison setting (Awenat et al., 2018; MacInnes et al., 2011). In addition, in this study we analyzed data from focus groups focused on reproductive healthcare in prison broadly, rather than contraception specifically. Had the focus groups been designed with an explicit focus on contraception, they may have yielded additional insights. Finally, we conducted only three focus groups in one facility, based on what was feasible and acceptable for the correctional authority and with study resources. Nonetheless this study contributes to an evolving picture of the experiences and perspectives of women who are incarcerated regarding contraception.

## Conclusions

Our findings constitute a meaningful contribution to research that characterizes and responds to the contraception-related healthcare needs of women in prisons in Canada and around the world, and suggest several opportunities to address needs. Firstly, our findings suggest that many women in prisons do not feel they have access to the information they need in order to make informed decisions about their contraceptive needs, indicating a potential role for enhanced contraception information in correctional facilities. Secondly, discussions expressing interests in future fertility indicate that reproductive health care in prison should include services related to contraception as well as preconception and parenting supports, to protect women’s right to have children as well as the right not to have children, recognizing the history of coercion and stigma often experienced by women in prisons who want to have children (Johnson, 2013; Winters and McLaughlin, 2019; Roth & Ainsworth, 2015). Finally, study participants described admission to and release from prison as points of vulnerability that put them at risk of losing access to care, suggesting points of focus to increase access to services and improve continuity of care between the prison and community setting. Implementation of these practical downstream interventions must be paired with an acknowledgement that access to reproductive dignity and justice for women who are incarcerated is fundamentally challenged by prison settings.

### Electronic supplementary material

Below is the link to the electronic supplementary material.

Supplementary Material 1

## Data Availability

The datasets used and/or analysed during the currentstudy are available from the corresponding author on reasonable request.

## Abbreviations

US: United States
IUD: Intrauterine device
DMPA: Depo-metroprogesterone acetate

## References

[CR1] Atabay, T. (2008). *Handbook for prison managers and policymakers on women and imprisonment*. UN.

[CR2] Awenat YF, Moore C, Gooding PA, Ulph F, Mirza A, Pratt D (2018). Improving the quality of prison research: A qualitative study of ex-offender service user involvement in prison suicide prevention research. Health Expectations.

[CR3] Barberet, R., & Jackson, C. (2017). UN rules for the treatment of women prisoners and non-custodial sanctions for women offenders (the Bangkok Rules): A gendered critique. *Papers: revista de sociologia*, *102*(2), 0215–230.Cannon, R., Madrigal, J. M., Feldman, E., Stempinski-Metoyer, K., Holloway, L., & Patel, A. (2018). Contraceptive needs among newly incarcerated women in a county jail in the United States. *International Journal of Prisoner Health*, *14*(4), 244–253. 10.1108/IJPH-08-2017-0036 30468113

[CR4] Cavanagh, A., Shamsheri, T., Shen, K., Gaber, J., Liauw, J., Vanstone, M., & Kouyoumdjian, F. (2022). Lived experiences of pregnancy and prison through a reproductive justice lens: A qualitative meta-synthesis. *Social Science & Medicine*, 115179. 10.1016/j.socscimed.2022.115179 35809528

[CR5] Clarke JG, Rosengard C, Rose JS, Hebert MR, Peipert J, Stein MD (2006). Improving birth control service utilization by offering services prerelease vs postincarceration. American Journal of Public Health.

[CR6] Johnson, C. G. (2013). Female inmates sterilized in California prisons without approval. *Center for investigative reporting*, *7*.

[CR7] Kajstura, A. (2018). *Women’s mass incarceration: The whole pie 2018*. Prison Policy Initiative.

[CR8] LaRochelle F, Castro C, Goldenson J, Tulsky JP, Cohan DL, Blumenthal PD, Sufrin CB (2012). Contraceptive use and barriers to access among newly arrested women. Journal of Correctional Health Care.

[CR10] Liauw J, Foran J, Dineley B, Costescu D, Kouyoumdjian FG (2016). The unmet contraceptive need of incarcerated women in Ontario. Journal of Obstetrics and Gynaecology Canada.

[CR9] Liauw, J., Jurgutis, J., Nouvet, E., Dineley, B., Kearney, H., Reaka, N., & Kouyoumdjian, F. (2021). Reproductive healthcare in prison: A qualitative study of women’s experiences and perspectives in Ontario, Canada. *PloS One*, 16(5), e0251853. 10.1371/journal.pone.0251853 PMC8130921 34003876

[CR11] Maillet, M. (2023). Correctional Investigator Releases Updated Findings on the State of Indigenous Corrections in Canada: National Indigenous Organizations Issue Statements of Support. *Office of the Correctional Investigator*, https://oci-bec.gc.ca/en/content/correctional-investigator-releases-updated-findings-state-indigenous-corrections*canada*.

[CR13] Malakieh, J. (2020). Table 4: Admissions to adult custody, by sex and by Indigenous identity and jurisdiction, 2018/2019. *Statistics Canada* https://www150.statcan.gc.ca/n1/pub/85-002-x/2020001/article/00016/tbl/tbl04-eng.htm.

[CR12] Malakieh, J. (2022). Adult and Youth Correctional Statistics in Canada, 2020/2021. *Statistics Canada* https://www150.statcan.gc.ca/n1/daily-quotidien/220420/dq220420c-eng.htm.

[CR14] O’Mahen HA, Flynn HA (2008). Preferences and perceived barriers to treatment for depression during the perinatal period. Journal of Women’s Health.

[CR15] Oswalt K, Hale GJ, Cropsey KL, Villalobos GC, Ivey SE, Matthews CA (2010). The contraceptive needs for STD protection among women in jail. Health Education & Behavior.

[CR16] Peart MS, Knittel AK (2020). Contraception need and available services among incarcerated women in the United States: A systematic review. Contraception and Reproductive Medicine.

[CR17] Pemberton S (2013). Enforcing gender: The constitution of sex and gender in prison regimes. Signs: Journal of Women in Culture and Society.

[CR18] Reitano, J. (2015). Adult Correctional Statistics in Canada, 2014/15. Statistics Canada. http://www.statcan.gc.ca/pub/85-002-x/2016001/article/14318-eng.htm.

[CR19] Ross, L., & Solinger, R. (2017). *Reproductive justice: An introduction* (Vol. 1). Univ of California.

[CR20] Roth R, Ainsworth SL (2015). If they hand you a paper, you sign it: A call to end the sterilization of women in prison. Hastings Women’s LJ.

[CR21] Saunders B (2018). Saturation in qualitative research: Exploring its conceptualization and operationalization. Quality & Quantity.

[CR22] Schonberg D, Bennett AH, Sufrin C, Karasz A, Gold M (2015). What women want: A qualitative study of contraception in jail. American Journal of Public Health.

[CR23] Singla, D. R., Lemberg-Pelly, S., Lawson, A., Zahedi, N., Thomas-Jacques, T., & Dennis, C. L. (2020). Implementing psychological interventions through nonspecialist providers and telemedicine in high-income countries: Qualitative study from a multistakeholder perspective. *JMIR Mental Health*, 7(8), e19271. 10.2196/19271 PMC7484770 32852281

[CR24] Sufrin C, Baird S, Clarke J, Feldman E (2017). Family planning services for incarcerated women: Models for filling an unmet need. International Journal of Prisoner Health.

[CR25] Winters DJ, McLaughlin AR (2020). Soft sterilization: Long-acting reversible contraceptives in the carceral state. Affilia.

