# Supplementary material for: “We wish we had the option”: a qualitative study of women’s perspectives and experiences with contraception in a provincial prison in Ontario, Canada
Source: Health Justice. 2024 Apr 12;12:15. doi: 10.1186/s40352-024-00269-6 (PMC11010403; doi:10.1186/s40352-024-00269-6)
Supplement: Supplementary file 1 — Supplementary Material 1 [file 40352_2024_269_MOESM1_ESM.docx]

**Supplementary Table 1. Focus Group Guide**

| General attitudes toward pregnancy | 1. Thinking back on your experiences and the experiences of other women who have spent time in jail or prison, do you think women are able to decide whether and when to become pregnant?    2. Do you think it is important for a woman to be able to control if she gets pregnant? Why or why not?    3. What has pregnancy meant to you now or in the past? (Prompt: What does it mean to you to be pregnant or have a baby?) |
| --- | --- |
| Attitudes toward contraception | 1. What methods of birth control, if any, have you tried? Have these methods worked for you? Would you use any of these methods of birth control again in the future? Why or why not?    2. Are there some methods of birth control you have heard about but think: ‘I don’t trust that’ or ‘that is not for me’?  Rephrase option: Are there methods of birth control you want to avoid? Why that or those method(s)?    3. Have you ever wanted a type of birth control but you could not get it? If yes: what was the form of birth control? Was that in jail or in the community? How did that make you feel? What did you do?    4. Are there times when you have chosen not to use birth control but also didn’t want to become pregnant? Are there any reasons you prefer not to use birth control?    5. Is birth control important to women while in jail? Why or why not?    6. In your experience, is access to birth control more or less of a concern after release? (Rephrase option: In your experience, is access to birth control more important to you while in jail or after release?) |
| Attitudes towards pregnancy in custody and after release | 1. What would it mean to be pregnant in custody? (Prompt: Suppose you had found out you were pregnant while in custody, how you would have reacted? What are some of the thoughts or considerations that would make you react in that way or feel that way?)    2. What would it mean for you to get pregnant soon after release, for example, within a year or two? |
| Barriers to accessing contraception | 1. In your view, why do women get pregnant when they are not planning to?    2. What are some difficulties women face in using birth control? |
| Strategies to improving access to effective contraception. | 1. What services would help women control whether or not they become pregnant?    2. What could we do for women in jail so they have better access to the birth control they want?    3. Why do you feel this would help women?    4. What could we do for women after they are released so they can have better access to birth control if they want it? |
| Wrap up | Is there anything else you would like to say about pregnancy or birth control? |
